# Coaching stroke survivors to persevere with practice: An observational behavioural mapping study

**DOI:** 10.1177/02692155241304340

**Published:** 2024-12-08

**Authors:** Bridee Neibling, Moira Smith, Ruth N Barker, Kathryn S Hayward

**Affiliations:** 1 College of Healthcare Sciences, 8001 James Cook University, Townsville, QLD, Australia; 2 Physiotherapy Department, Townsville University Hospital, Townsville, QLD, Australia; 3 The Cairns Institute, 8001 James Cook University, Cairns, QLD, Australia; 4 Departments of Physiotherapy and Medicine, 2281 University of Melbourne - Parkville Campus, Melbourne, Australia

**Keywords:** Stroke rehabilitation, perseverance, coaching, behavioural mapping

## Abstract

**Objective:**

To quantitatively describe therapists’ use of coaching with stroke survivors, in a hospital-based rehabilitation setting, to promote perseverance with longer-term practice.

**Design:**

Prospective observational behavioural mapping study.

**Setting:**

Rehabilitation unit of a regional public hospital in Queensland, Australia.

**Main measures:**

A custom-designed behavioural mapping tool was used to collect rehabilitation session contextual data and therapists' use of coaching. Data were captured in 3-minute epochs for a maximum of 30 minutes. Data were analysed using descriptive statistics.

**Results:**

Thirty-six rehabilitation sessions, including 34 participants (therapists *n* = 22, stroke survivors *n* = 12) were observed. Rehabilitation sessions were mostly inpatient (*n* = 33, 91.7%), one-on-one (*n* = 30, 83.3%), and conducted in the physiotherapy (*n* = 160, 45.5%) or occupational therapy (*n* = 155, 44.0%) gym. Strategies to promote perseverance were used in 76.7% (*n* = 267) of observed epochs. The most frequently used strategy was *monitoring the quality of practice* and the least frequently used strategy was *utilising a support person to facilitate practice.*

**Conclusion:**

Coaching that may promote perseverance with practice was regularly used by therapists during hospital-based rehabilitation sessions. Coaching that may enable longer-term perseverance beyond a therapist-dependent rehabilitation model was less commonly observed.

## Introduction

Stroke is among the leading causes of disability in Australia.^1^ In 2020, more than 445,000 Australians were living with the effects of stroke.^2^ Stroke survivors recognise that longer-term perseverance with practice is key to functional recovery, reducing disability and improving quality of life.^3–5^ Yet, attention to factors that may promote perseverance appears to be neglected during hospital-based rehabilitation.^5^ This can leave stroke survivors feeling ill-equipped when transitioning from hospital- to home-based rehabilitation.^5^

To persevere with longer-term practice, stroke survivors report needing opportunities to engage in the personalised, goal-directed practice, that is appropriately challenging, fits into their everyday life, and produces meaningful outcomes.^3,5,6^ To facilitate practice, access to appropriate support, therapy, and equipment is imperative.^3,5,6^ Stroke survivors need training to understand what to practice, how to monitor and modify practice, and how to monitor progress.^5^ Strategies targeting the aforementioned domains need to be considered early in the rehabilitation process, and continue throughout recovery, to promote perseverance with longer-term practice.^5^

Coaching to promote perseverance with longer-term practice can form part of scheduled hospital-based rehabilitation sessions, and includes strategies defined through qualitative investigations with stroke survivors^3,5,6^ and incorporation of motor learning principles.^7–12^ Stroke survivors and their carers should be involved in the co-creation of meaningful, person-centred, short- and long-term goals to drive recovery.^13^ Stroke survivors’ problem-solving skills and self-efficacy for practice should be enriched through the provision of appropriate instructions/cues and feedback,^7–12^ education on how to practice and monitor progress,^8,11,12^ and instructions on how to modify practice to ensure it is appropriately challenging.^14^ Involving carers in therapy should mitigate some of the challenges associated with the outside of therapy practice (e.g. practice setup, safety concerns, supervision etc.), improve tailoring of rehabilitation to the stroke survivors needs, and allow carers to feel involved in the rehabilitation process and validated in their support role.^15^ Prescription of hospital-based independent and carer-mediated practice should build stroke survivors and carers' capability and opportunity for longer-term practice.^5^ Quantitative measurement of coaching within the context of hospital-based rehabilitation sessions is lacking. Thus, the purpose of this study was to quantitatively describe therapists’ use of coaching with stroke survivors, in a hospital-based rehabilitation setting, to promote perseverance with longer-term practice.

## Methods

### Design and setting

This prospective observational behavioural mapping study was performed in the rehabilitation unit of a regional public hospital in Queensland, Australia. Behavioural mapping has been used extensively in stroke research.^16–20^ It enables frequent recording of therapist and patient behaviour and environmental variables in pre-determined categories over small epochs of time.^16,19,21,22^ Behavioural mapping was chosen to provide an objective assessment of therapists’ behaviour and minimise the risk of bias which could have occurred with a self-report tool.^16^

The rehabilitation unit used in this study provides both inpatient and outpatient rehabilitation services to people with a range of neurological, orthopaedic and reconditioning diagnoses. Twenty-five percent of admissions annually are for people with disabling consequences of stroke. The 45-bed inpatient ward has 21.6 full-time equivalents of combined physiotherapist, occupational therapist and allied health assistant staff. The outpatient service is staffed by 1 full-time equivalent physiotherapist and 0.6 full-time equivalent occupational therapist.

The study was conducted in accordance with the Declaration of Helsinki and approved by the Townsville Hospital and Health Service (HREC/QTHS/80488), and James Cook University (HREC/H8884), Human Research Ethics Committees. All participants provided written informed consent.

### Participants

Participants were therapists (physiotherapists, occupational therapists, and allied health assistants) providing, and stroke survivors receiving, rehabilitation sessions. Therapists were eligible to participate if they were (1)  ≥ 18 years of age; (2) working as a physiotherapist, occupational therapist, or allied health assistant; and (3) providing rehabilitation sessions to stroke survivors participating in the study. Stroke survivors were eligible if they were (1)  ≥ 18 years of age; (2) had a diagnosis of haemorrhagic or ischaemic stroke in the last 12 months; (3) had an upper or lower limb deficit resulting from a stroke that required rehabilitation; (4) were actively participating in hospital-based physiotherapy and/or occupational therapy rehabilitation sessions; (5) had experienced > 1 week of rehabilitation sessions; (6) were able to communicate (either verbally, written or with augmented communication devices); and (7) were able to provide informed consent. Therapists and stroke survivors who met the eligibility criteria were recruited according to their availability at the time of data collection (i.e. convenience sampling).^23^

### Data Collection

Therapist demographic data were collected at recruitment and included: age, sex, profession, and years of professional experience. Stroke survivor demographic data were collected at recruitment and included: age, sex, date of stroke, type of stroke, affected side, and stroke severity according to the National Institute of Health Stroke Scale (i.e. mild (< 8), moderate (8–16), and severe (> 16)).^24,25^

Observational data were collected during physiotherapy-, occupational therapy-, and allied health assistant-led rehabilitation sessions. The behavioural mapping tool used was custom-designed to collect coaching data not captured by previous mapping tools.^16–20^ Firstly, a tool prototype was developed. To determine tool components, the lead investigator: (1) reviewed existing literature on behavioural mapping in a rehabilitation setting to determine the method of observation and contextual information required^16–20^; (2) reviewed qualitative reports of stroke survivors’ perspectives of factors influencing their ability to persevere with longer-term practice^3,5,6^ and translated this into observable strategies; and (3) reviewed principles of motor learning defined in literature.^7–12^ The initial prototype was then refined through 10 iterative discussions with the wider research team. Secondly, the tool was piloted in a hospital-based rehabilitation setting to ensure that it appropriately captured the context and content of rehabilitation sessions and was practical to apply (e.g. timely to complete and minimised disruption to rehabilitation sessions). The lead investigator piloted the tool during seven rehabilitation sessions involving seven therapists and three stroke survivors. Field notes were used to capture lessons learned during piloting, which were discussed with the research team to refine the tool. The final version (Supplemental Material 1) collected data regarding: (1) context; (2) strategies to promote perseverance; and (3) incorporation of motor learning principles (Table 1).

**Table 1.** Data collected via a custom-designed behavioural mapping tool.

Context	Location People present Rest Treatment target Treatment focus
Strategies to promote perseverance	Setting goals Setting-up practice Monitoring the quantity of practice Monitoring the quality of practice Modifying practice Monitoring progress Exercises to complete independently during therapy Exercises to complete independently outside of therapy Utilising a support person to facilitate practice Utilising everyday equipment to facilitate practice Fitting practice into everyday life
Motor learning principles	Goals Instruction/cueing Feedback Performance measurement

All behavioural mapping was conducted by the lead investigator. The intention was to observe up to five rehabilitation sessions per stroke survivor, including at least one occupational therapist-led and one physiotherapist-led session, ± an allied health assistant-led session. Rehabilitation sessions were continuously observed for the first 30 minutes for timeframe homogeneity. Session data were documented in 3-minute epochs. If an activity precluded direct observation (e.g. toileting), the observer attempted to retrospectively estimate the data with the assistance of the therapist and stroke survivor. If a rehabilitation session ended prior to 30 minutes (e.g. all practice completed, patient taken off the ward for scan), the remaining epochs were classed as ‘missing’ data. The proportion of epochs and time, along with a reason for missing data were documented.

### Data Analysis

All mapping data were manually recorded and then transcribed into a Microsoft Excel Spreadsheet for analysis by the lead investigator. Data were analysed descriptively as sample size did not allow for inferential statistics. Missing data were not included in the analysis.

Participant demographic data were expressed using median (interquartile range) for continuous variables and counts (proportion) for categorical variables. Contextual data were expressed in terms of count (*n*) and relative frequency (%) for session type, location, people present, rest, treatment target and treatment focus. The number of rehabilitation sessions observed per participant was expressed using the median and interquartile range. Strategies to promote perseverance were expressed in terms of the count (*n*) and frequency of epochs (%) in which they were used, and the relative frequency (%) of use for each strategy. Motor learning principles were expressed in terms of the count (*n*) and frequency of epochs (%) in which they were incorporated, and relative frequency (%) according to grouped type (e.g. degree of collaboration in goal setting).

## Results

Thirty-four participants were observed (therapists *n* = 22 and stroke survivors *n* = 12). Table 2 details participant demographics. In brief, therapists were mostly female with less than 3 years of professional experience. Stroke survivors were mostly male, had a mild or moderate ischaemic stroke, and were in the early subacute phase of recovery^26^ at recruitment.

**Table 2.** Participant demographics.

Therapists (*n* = 22)	
Age – years, median (IQR)	28.5 (24.8, 36.0)
Sex, *n* (%)	
Male	7 (31.8%)
Female	15 (68.2%)
Profession, *n* (%)	
Physiotherapist	7 (31.8%)
Occupational therapist	5 (22.7%)
Allied Health Assistant	10 (45.5%)
Professional experience – years, median (IQR)	2.9 (1.6, 6.6)
Stroke survivors (*n* = 12)	
Age (years), median (IQR)	66.5 (56.8, 70.8)
Sex, *n* (%)	
Male	8 (66.7%)
Female	4 (33.3%)
Type of stroke, *n* (%)	
Ischaemic	11 (91.7%)
Haemorrhagic	1 (8.3%)
National Institute of Health Stroke Scale – Total, *n* (%)	
Mild (< 8)	7 (58.3%)
Moderate (8–16)	5 (41.7%)
Severe (> 16)	0 (0.0%)
National Institute of Health Stroke Scale – Sensory Impairment, *n* (%)	7 (58.3%)
National Institute of Health Stroke Scale – Aphasia, *n* (%)	3 (25.0%)
National Institute of Health Stroke Scale – Extinction and Inattention, *n* (%)	2 (16.7%)
Time since stroke at baseline assessment – days, median (IQR)	56.0 (41.8, 77.3)
Time since stroke at observation date (*n* = 35) – days, median (IQR)	67.0 (47.8, 92.0)

*Note:* IQR: interquartile range; *n*: number of participants.

Thirty-six rehabilitation sessions, comprising 348 epochs, were observed between July 2023 and March 2024. Data were missing for 12 epochs across five rehabilitation sessions: three rehabilitation sessions were < 30 minutes in duration resulting in four missing epochs, and two sessions were ceased by the stroke survivor resulting in eight missing epochs.

Contextual factors noted during observed epochs are expressed in detail in Table 3. In short, rehabilitation sessions were mostly inpatient, one-on-one, and led by an even distribution of physiotherapists, occupational therapists, and allied health assistants. Sessions were mostly conducted in either the physiotherapy or occupational therapy gym, and evenly targeted upper and lower limb practice of impairment or activity.^27^

**Table 3.** Contextual factors noted during observed epochs.

Session type (*n* = 36)		*n* (%)
Discipline lead	Physiotherapy	10 (27.8%)
	Occupational therapy	9 (25.0%)
	Allied Health Assistant (physiotherapy)	10 (27.8%)
	Allied Health Assistant (occupational therapy)	7 (19.4%)
Type	Individual	30 (83.3%)
	Group	6 (16.7%)
Setting	Inpatient	33 (91.7%)
	Outpatient	3 (8.3%)
Location (*n* = 352)^a^		*n* (%)
Physiotherapy gym		160 (45.5%)
Occupational therapy gym		155 (44.0%)
Outpatient rehabilitation gym		20 (5.7%)
Bedside		11 (3.1%)
Outside (e.g. garden and carpark)		4 (1.1%)
Bathroom/toilet		1 (0.3%)
Corridor		1 (0.3%)
Dining area, lounge area, quiet/cognitive room, consult room		0 (0.0%)
People present (*n* = 470)^a^		*n* (%)
Allied health assistant		165 (35.1%)
Physiotherapist		107 (22.8%)
Occupational therapist		84 (17.9%)
Visitor		78 (16.6%)
Occupational therapy student		16 (3.4%)
Other health professional		12 (2.6%)
Alone		8 (1.7%)
Physiotherapy student		0 (0.0%)
Rest (*n* = 348)		*n* (%)
Nil		171 (49.1%)
< 1 minute		112 (32.2%)
1–2 minutes		41 (11.8%)
> 2 minutes		24 (6.9%)
Treatment target (*n* = 355)^a^		*n* (%)
Upper limb		180 (50.7%)
Lower limb		146 (41.1%)
Nil		26 (7.3%)
Other		3 (0.8%)
Treatment focus (*n* = 354)^a^		*n* (%)
Impairment^27^		223 (63.0%)
Activity^27^		95 (26.8%)
Nil		26 (7.3%)
Participation^27^		10 (2.8%)

*Note:* IQR: interquartile range; *n*: total number of observations.

a Possibility of more than one observation per epoch (e.g. therapy conducted in more than one location during a 3-minute epoch).

Strategies to promote perseverance were used in 76.7% (*n* = 267) of observed epochs. Table 4 details strategies and their relative frequency of use.

**Table 4.** Strategies to promote perseverance used during observed epochs.

Strategies (*n* = 467)^a^	*n* (%)
Monitoring the quality of practice	128 (27.4%)
Setting-up practice	58 (12.4%)
Monitoring the quantity of practice	52 (11.1%)
Exercises to complete independently during therapy	50 (10.7%)
Monitoring progress	45 (9.6%)
Utilising everyday equipment to facilitate practice	35 (7.5%)
Other (e.g. explanation of neuroplasticity)	26 (5.6%)
Setting goals	20 (4.3%)
Modifying practice	20 (4.3%)
Exercises to complete independently outside of therapy	14 (3.0%)
Fitting practice into everyday life	13 (2.8%)
Utilising a support person to facilitate practice	6 (1.3%)

*Note: n*: total number of observations.

a Possibility of more than one observation per epoch (e.g. more than one strategy used during a 3-minute epoch).

Motor learning principles incorporated are detailed in Table 5. Goals were set in 51.7% (*n* = 180) of observed epochs. Most goals were non-collaborative, therapist-centred, and session focused. Instructions and cues were provided in 81.0% (*n* = 282) of observed epochs. Instructions were predominantly verbal, with an even distribution of internal and external foci, and supported by verbal and visual cues. Feedback was provided in 77.9% (*n* = 271) of observed epochs, consisting mostly of general encouragement delivered concurrently with therapy. Where knowledge of performance and results feedback were stipulated, there was an even distribution of internal and external foci. Performance was measured in 85.1% (*n* = 296) of observed epochs and was largely a count-based metric.

**Table 5.** Motor learning principles incorporated during observed epochs.

Goals		*n* (%)
Goal focus (*n* = 181)^a^	Session goal	163 (90.1%)
	Short-term goal	14 (7.7%)
	Long-term goal	4 (2.2%)
Goal type (*n* = 181)^a^	Therapist-centred	162 (89.5%)
	Person-centred	19 (10.5%)
Collaboration (*n* = 187)^a^	Non-collaborative	143 (76.5%)
	Collaborative	44 (23.5%)
Instructions/cues		**n (%)**
Instruction type (*n* = 292)^a^	Verbal	220 (75.3%)
	Modelling/demonstration	66 (22.6%)
	Written	5 (1.7%)
	Video	1 (0.3%)
Instruction focus (*n* = 209)^a^	Internal	111 (53.1%)
	External	98 (46.9%)
Cue type (*n* = 222)^a^	Verbal	111 (50.0%)
	Visual	101 (45.5%)
	Tactile	9 (4.1%)
	Auditory	1 (0.5%)
Feedback		***n* (%)**
Feedback type (*n* = 448)^a^	General encouragement	206 (46.0%)
	Knowledge of results	131 (29.2%)
	Knowledge of performance – prescriptive	56 (12.5%)
	Knowledge of performance – descriptive	55 (12.3%)
Feedback focus (*n* = 47)^a^	Internal	24 (51.1%)
	External	23 (48.9%)
Timing (*n* = 233)^a^	Concurrent	207 (88.8%)
	Delayed	26 (11.2%)
Performance measurement		***n* (%)**
Type (*n* = 499)^a^	Count-based	249 (49.9%)
	Range of movement-based	85 (17.0%)
	Distance-based	57 (11.4%)
	Degree of task completion	36 (7.2%)
	Height-based	31 (6.2%)
	Time-based	20 (4.0%)
	Weight-based	20 (4.0%)
	Other	1 (0.2%)

*Note: n*: total number of observations.

a Possibility of more than one observation per epoch (e.g. more than one type of instruction/cue used during a 3-minute epoch).

## Discussion

This study describes therapists’ use of coaching with stroke survivors, in a hospital-based rehabilitation setting, using direct behavioural observation. The results highlight that, during rehabilitation sessions, therapists are frequently using coaching that may promote perseverance. The most used strategies – *monitoring the quality* and *quantity of practice* and *progress*, *setting up practice,* and prescribing *exercises to complete independently during therapy* – align with evidence and best practice guidelines to encourage accurate and intensive task-specific practice.^5,13,28,29^ However, this highly structured practice may increase therapist-dependence. The least used strategies – *setting goals, modifying practice, exercises to complete independently outside of therapy*, *fitting practice into everyday life* and *utilisation of support people to facilitate practice* – may decrease therapist-dependence and enhance non-therapist-led practice opportunities. Importantly, these strategies could better prepare stroke survivors and their carers for the transition to home-based practice, ultimately promoting longer-term perseverance.^5,30,31^

In this study, goals were set in only half of the observed epochs, and were largely therapist-centred, non-collaborative, session-focused goals. The challenge of setting person-centred, collaborative, short- and long-term goals in an inpatient setting is well recognised.^32–36^ During inpatient rehabilitation, goal setting is largely driven by therapists who need to fulfil a professional (e.g. ensuring independence with activities of daily living) and organisational (e.g. timely discharge) obligation.^32,33,35^ This leads to the creation of ‘privileged goals’ which are physical in nature, reflect the process of rehabilitation, and are achievable in the inpatient context.^33^ To enhance stroke survivors’ motivation to persevere with longer-term practice, therapists must prioritise the co-creation of meaningful, person-centred, long-term goals, and measure progress towards these in a hospital-based setting.^5^

This study observed that therapists were proactive in teaching stroke survivors how to *monitor the quality* and *quantity of practice* and *monitor progress* but independently led the decision-making process for *modification of practice*. To maximise therapeutic engagement and functional recovery, therapists must focus on collaborating with stroke survivors and their carers to pitch practice at the optimal challenge level.^14^ Practice that is not sufficiently challenging results in minimal learning and functional change, increasing stroke survivors’ boredom and disengagement from therapy.^14^ Practice that is too challenging causes poor performance and safety concerns, prompting frustration and decreased confidence which makes recovery seem unrealistic, ultimately resulting in stroke survivors abandoning rehabilitation.^14^

Therapists’ provision of information regarding *exercises to complete independently outside of therapy* and *utilisation of support people to facilitate practice* were infrequently observed in this study. There was limited use of written or video-based instructions/cues for practice and minimal engagement of support people attending rehabilitation sessions. This paucity of information provided about independent and carer-mediated practice extends to the findings of previous research about barriers to activity outside of scheduled therapy.^31^ Coaching that increases carer involvement and out-of-sessions practice should be prioritised, recognising that this may improve function and optimise quality of life outcomes for stroke survivors both during and following hospital-based rehabilitation.^37,38^

Messaging around *fitting practice into everyday life* needs further consideration, based on this study's findings. Although stroke survivors participate in activities of daily living outside of therapy, this is not consistently regarded as ‘practice’.^3,5^ Therapists and stroke survivors place a higher value on structured practice compared with incidental activity.^5,30^ *Fitting practice into everyday life* is not simply about ensuring stroke survivors complete structured practice every day.^5^ To drive habitual changes that promote longer-term perseverance with practice and post-stroke recovery, stroke survivors and therapists need to consider a cultural shift whereby incidental activity (e.g. preparing breakfast with encouragement to use both arms) is considered opportunistic practice and recognised as valuable therapy.^5,30^ Accordingly, opportunistic coaching needs to be recognised as valuable coaching.

The behavioural mapping tool used in this study was custom-designed to include best-practice evidence, grounded in the perspectives of stroke survivors, and piloted in a rehabilitation unit to ensure content was appropriate.^21^ Specifics of coaching being recorded were not disclosed to the participants to limit alteration of their behaviour during observation. While the reliability and validity of the mapping tool were not formally tested, a single researcher completed all data collection to ensure a consistent approach to data recording was applied across observations. A 30-minute continuous behavioural mapping approach meant that coaching could be captured even if it were fleeting,^21^ however it also meant only part of some rehabilitation sessions were observed. Consequently, some coaching may have been missed (e.g. therapists prescribing independent practice at the end of a session, or therapists providing opportunistic coaching while accompanying the stroke survivor to their next therapy session). The results are reflective of the sample included (i.e. mild-moderate stroke survivors receiving inpatient rehabilitation at a single regional centre). The tool is potentially transferable to other rehabilitation environments, with context-specific variables adapted on a per-unit basis. A larger sample size would allow for inferential statistics and the opportunity to explore relationships between variables (e.g. coaching according to therapist experience).

This behavioural mapping tool offers objective insight into therapists’ provision of coaching to promote perseverance with practice. It has the potential to be used for service-based audits or self-assessment and reflective practice. Further research is required to capture end-of-session content and out-of-session opportunistic coaching (e.g. from nurses during showering and from carers during family visits). Research to confirm the validity and reliability of the tool, explain observed patterns of therapist behaviour, and develop interventions that address observed gaps in coaching is also required.

**Clinical messages**

- Coaching most frequently used during hospital-based rehabilitation sessions, encourages stroke survivor dependence on therapists.
- Increased attention to coaching that prepares stroke survivors for the transition to non-therapist-led practice could promote longer-term perseverance.

## Supplemental Material

sj-pdf-1-cre-10.1177_02692155241304340 - Supplemental material for Coaching stroke survivors to persevere with practice: An observational behavioural mapping study Supplemental material, sj-pdf-1-cre-10.1177_02692155241304340 for Coaching stroke survivors to persevere with practice: An observational behavioural mapping study by Bridee Neibling, Moira Smith, Ruth N Barker and Kathryn S Hayward in Clinical Rehabilitation
