# Supplementary material for: Coaching stroke survivors to persevere with practice: An observational behavioural mapping study
Source: Clin Rehabil. 2024 Dec 8;39(3):410–20. doi: 10.1177/02692155241304340 (PMC11927032; doi:10.1177/02692155241304340)
Supplement: sj-pdf-1-cre-10.1177_02692155241304340 - Supplemental material for Coaching stroke survivors to persevere with practice: An observational behavioural mapping study [file sj-pdf-1-cre-10.1177_02692155241304340.pdf]

Supplemental Material 1. Behavioural mapping tool

**Stroke Survivor ID:**	**Date:**	**Qualitative Reflecion/Field Notes:**
**Therapist ID:**	**Session Type:**	

Time	Location (1-12)	People Present (1-8)	Rest (1-4)	Treatment Target (1-4)	Treatment Focus (1-4)	Perseverance Coaching Strategies (1-13)	Goals (1-9)	Instruction/ Cueing (1-13)	Feedback (1-11)	Performance Measurement (1-9)
3										
6										
9										
12										
15										
18										
21										
24										
27										
30										

Location	People Present	Rest	Treatment Target	Treatment Focus	Perseverance Coaching Strategies	Goals	Instruction/ Cueing	Feedback	Performance Measurement
1 - Bedside	1 - Physiotherapist	1 - Nil	1 - Upper Limb	1 - Impairment	1 - Setting goals	1 - Long-term goal	1 - Goal-oriented instruction	1 - Goal-oriented feedback	1 - Time-based
2 - Bathroom/Toilet	2 - Occupational Therapist	2 - <1 minute	2 - Lower Limb	2 - Activity	2 - Setting up practice	2 - Short-term goal	2 - Verbal instruction	2 - Knowledge of performance - descriptive	2 - Count-based
3 - Corridor	3 - Allied Health Assistant	3 - 1-2 minutes	3 - Other	3 - Participation	3 - Monitoring the quantity of practice	3 - Session goal	3 - Written instruction	3 - Knowledge of performance - prescriptive	3 - ROM-based
4 - Dining Area	4 - Visitor	4 - > 2 minutes	4 - Nil	4 - Nil	4 - Monitoring the quality of practice	4 - Person-centred	4 - Video instruction	4 - Knowledge of results	4 - Weight-based
5 - Lounge Area	5 - Physiotherapy Student				5 - Modifying practice	5 - Therapist centred	5 - Internal focus	5 - Internal focus	5 - Height-based
6 - Occupational Therapy Gym	6 - Occupational Therapy Student				6 - Monitoring progress	6 - Collaborative	6 - External focus	6 - External focus	6 - Distance-based
7 - Physiotherapy Gym	7 - Other Health Professional				7 - Exercises to complete independently during therapy	7 - Non-collaborative	7 - Modelling/ demonstration	7 - Concurrent	7 - Degree of task completion
8 - Outpatient Rehabilitation Gym	8 - Alone				8 - Exercises to complete independently outside of therapy	8 - Other	8 - Verbal cue	8 - Delayed	8 - Other
9 - Quiet/Cog Room					9 - Utilising a support person to facilitate practice	9 - No goals set with patient	9 - Visual cue	9 - Encouragement	9 - No performance measurement
10 - Consult Room					10 - Utilising everyday equipment to facilitate practice		10 - Auditory cue	10 - Other	
11 - Outside					11 - Fitting practice into everyday life		11 - Tactile cue	11 - No feedback provided	
12 - Other					12 - Other		12 - Other		
					13 - No independence in practice fostered		13 - No instruction/ cueing utilised		
